# Simultaneous bilateral native nephrectomy by retroperitoneal approach

**DOI:** 10.1590/S1677-5538.IBJU.2018.0435

**Published:** 2020-03-12

**Authors:** Piotr Jarzemski, Sławomir Listopadzki, Piotr Słupski, Marcin Jarzemski, Bartosz Brzoszczyk

**Affiliations:** 1 Jan Biziel University Hospital in Bydgoszcz Nicolaus Copernicus University in Torun Collegium Medicum in Bydgoszcz Poland Department of Urology, Jan Biziel University Hospital in Bydgoszcz, Nicolaus Copernicus University in Torun, Collegium Medicum in Bydgoszcz, Poland

**Keywords:** Nephrectomy, Laparoscopy, Kidney

## Abstract

The indication for simultaneous bilateral native nephrectomy and the choice of surgical technique is of key importance, as these patients are burdened with a large comorbidity. The paper reports our experience of seven successful and completed simultaneous bilateral native nephrectomy procedures with retroperitoneal approach in the patient’s flank position. Seven patients (mean age 34), were indicated for the removal of both kidneys before the planned transplant. Six patients underwent haemodialysis from 48 to 84 months, and one underwent peritoneal dialysis for 60 months. Two patients had undergone graftectomy. The indications were chronic infection or hypertension. The length of the kidneys ranged from 5.8 to 10cm. All procedures were performed by the laparoscopic technique with retroperitoneal approach, with the patient in the flank position. Three trocars were used on each side. The retroperitoneal space created did not require balloon dilatation. The kidneys were removed through the 10mm trocar hole after splitting. The duration of the procedure ranged from 150 to 240 minutes, average 139 minutes and blood loss ranged from100 to 250mL, average 142mL. There were no complications. In 6 patients, the postoperative dialysis was performed at zero-day. One patient continued peritoneal dialysis. Patients were discharged on the 2nd day, except one with peritoneal dialysis, who was discharged on the 3rd day. Retroperitoneal laparoscopic bilateral native nephrectomy is a safe and effective technique, and it can be considered as an ideal approach for native nephrectomy. It allows for the preservation of peritoneal integrity and vessels for future vascular access.

## INTRODUCTION

In patients treated with renal replacement therapy, including those subjected to dialysis, numerous organ related complications, including kidney failure, are observed. Failures of treatment of complications within the organs by conservative methods lead to the implementation of treatment methods to remove the involved organ. The decision to undertake surgical treatment and the choice of surgical technique are of key importance, as these patients have a large comorbidity burden, which makes them particularly susceptible to complications during the postoperative period ( 1 ). Numerous methods of treatment during bilateral nephrectomy are particularly attractive for patients with high comorbidities, including laparoscopic nephrectomy. The procedure of bilateral laparoscopic nephrectomy was first described in 1994 by Bales et al., in two patients qualified for transplantation ( 2 ). In both cases, transperitoneal access was used. The use of the laparoscopic technique results in a shorter time of hospitalization and convalescence ( 3 - 5 ).

This advantage is extremely important for patients undergoing dialysis because it shortens the time between kidney removal and transplantation. The next stage in the development of surgical techniques was the removal of native kidney through the retroperitoneal approach, omitting the peritoneal cavity ( 4 - 7 ). The choice of access, patient placement and operative technique depends on the operator’s preferences. Compared to transperitoneal laparoscopic access, there appear to be several advantages to the retroperitoneoscopic approach for benign kidney disease. These advantages include ease of kidney access by developing the existing potential retroperitoneal space and avoidance of the transperitoneal approach with the resultant reduced risk of injury to and interference from intra-abdominal organs.

This article reports our experience of seven successful and completed simultaneous bilateral native nephrectomy procedures with retroperitoneal approach in the patient’s flank position.

## MATERIALS AND METHODS

The study consisted of 7 patients, including 4 men and 3 women between the ages of 20 and 68 (mean 34 years). All patients were undergoing long-term dialysis. Six patients underwent haemodialysis every 2 days, and one patient used peritoneal dialysis. Two patients had undergone kidney transplants and graftectomies due to rejection of the transplanted kidney. Patients were qualified for kidney removal by nephrologists if they had recurrent urinary tract infections and hypertension before the planned kidney transplant ( Table-1 ).

**Table 1:** Characteristics of patients enrolled in the study.

No.	Age (years)	Gender	Dimensions of the right kidney	Dimensions of the left kidney	Dialysis type and time (months)	Indications for nephrectomy
1	30	M	10.0 x 3.2 cm	9.6 x 3.0 cm	Haemodialysis 84 months	Chronic urinary tract infection; bilateral renal calculi
2	34	M	8.2 x 3.1 cm	9.7 x 4.5 cm	Haemodialysis 48 months	Chronic urinary tract infection; bilateral staghorn calculi
3	26	F	7.5 x 3.0 cm	6.6 x 3.2 cm	Peritoneal dialysis 60 months	Chronic urinary tract infection; bilateral vesicoureteral reflux.
4	25	M	6.4 x 3.0 cm	8.1 x 3.2 cm	Haemodialysis 72 months	Hypertension; glomerulonephritis
5	28	M	7.0 x 3.5 cm	7.0 x 3.4 cm	Haemodialysis 84 months. After transplantation and graftectomy in 2008.	Hypertension; distal renal tubular acidosis
6	28	F	5.8 x 2.6 cm	6.0 x 2.4 cm	Haemodialysis 72 months	Chronic urinary tract infection; bilateral vesicoureteral reflux.
7	68	F	10.0 x 4.4 cm	17.0 x 5.8 cm	Haemodialysis 84 months. After transplantation and graftectomy in 2010.	Chronic urinary tract infection; bilateral staghorn calculi

Before the operation, the following routine laboratory tests were performed: morphology, ionogram, creatinine, urea, prothrombin time, international normalized ratio (INR) and activated partial thromboplastin time, (APTT), ultrasound was also obtained.

On the day before the procedure, the patients underwent dialysis during the second shift in the evening. The operations were carried out the following day, early in the morning. After the procedure, all patients were transferred to the intensive care unit for monitoring of their vital signs. None patients required blood transfusions. In the zero-day period in the evening, all patients underwent hemodialysis except for one patient, who was continuing peritoneal dialysis.

**Figure 1:**
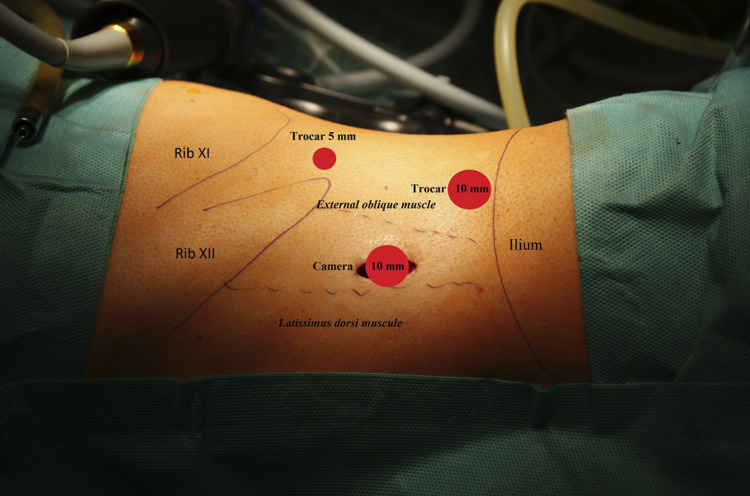
Scheme of patient's position with marked trocar sites. Patient placed in the left flank position during removal of the right kidney.

All treatments were performed by a retroperitoneoscopic technique. Patients were placed in the flank position, on their side, as for classical retroperitoneal surgery. First, the right kidney was removed, because in our opinion it is more difficult. Then, after transferring the patient to the opposite side, the left kidney was removed. The treatments started with a 1cm skin incision in the upper lumbar triangle. The retroperitoneal space was created only with the trocar and optics and did not require balloon dilatation of the retroperitoneal space, which was not routinely used. Optics with an angle of inclination of 30 degrees and a diameter of 10mm were used. After introducing the first trocar with the optics and performing insufflation to 12mmHg, additional trocars were inserted under visualisation control: a 10mm trocar over the iliac plate for the right hand of the operator and 5mm under the XI rib for the left hand. Three trocars (Karl Storz SE & Co. KG, Germany) were used, 2 x 10mm and 1 x 5mm on each side. Placement of the trocars in our patients is shown in Figure-1 .

All treatments were carried out in the same way. Preparation was started from visualization of the kidney cavity. First, the renal artery and vein were located. Artery and the vein were closed using hem-o-loc clips (Hem-o-lok® Ligation System, Teleflex Incorporated Earnings). After cutting the vessels, the ureter was visualized, clipped and cut. The kidney was inserted into the endoscopic bag and, after fragmentation with straight Kocher’s forceps, and removed through a 1cm hole after the 10mm trocar. A drain was left in the retroperitoneal space.

After removal of the right kidney, the patient was transferred to the opposite side, and the opposite kidney was removed in the same manner. Procedures on the left side were more difficult due to the presence of gas bubbles in the adipose tissue that filled the retroperitoneal space. The amount of gas was not very large, however, it slightly changed the anatomical conditions and the insight into the retroperitoneal space. The adipose tissue was loose and harder to dissect.

## RESULTS

The results are presented in Table-2 . There were no intra- or postoperative complications in any of the patients. We were not forced to convert the treatment to open surgery in any patients. In six patients, the first postoperative dialysis was performed in the zero-day period in the evening, and it was performed on the next one in the last patient. One patient continued peritoneal dialysis only during the entire postoperative period, without any staining of the dialysis fluid. Patients were discharged on the 2nd postoperative day, except for the peritoneal dialysis patient who was discharged on the 3rd day. All kidneys were morcellated and removed in fragments through the 10mm trocar hole.

**Table 2:** The results of the nephrectomy.

No.	Duration of the procedure	Loss of blood	Dialysis	Motor activity	Nutrition	Hospital stay
1	180 min	100 mL	Day: 0 and 1	1st day	1st day	2 days
2	240 min	160 mL	Day: 0 and 1	1st day	1st day	3 days
3	150 min	120 mL	Day: 0 peritoneal dialysis	1st day	2nd day	3 days
4	220 min	100 mL	Day: 0 and 1	1st day	1st day	2 days
5	160 min	150 mL	Day: 0 and 1	1st day	1st day	2 days
6	180 min	120 mL	Day: 0 and 1	1st day	1st day	2 days
7	240 min	250 mL	Day: 0 and 1	1st day	1st day	2 days

Postoperative pathomorphological assessments showed that the microscopic picture of the kidneys was dominated by pulp atrophy, glomerulosclerosis and the proliferation of connective tissue, which are features of chronic pyelonephritis.

## DISCUSSION

Retroperitoneoscopic nephrectomy is a standard technique for kidney removal in the case of benign non-functioning kidneys ( 8 ). The advantage of retroperitoneal access is surgery without the need to violate the peritoneal cavity. An inconvenience of retroperitoneal access, in the patient’s position on the side, in the case of simultaneous removal of both kidneys is the need to change the position of the patient during the procedure. However, this element could be omitted. Operations that include the removal of both kidneys from a retroperitoneal approach in the patient’s prone position are described and do not require a change of the patient’s position ( 9 - 11 ).

The indications for surgery in our patients, as reported by other authors, were recurrent urinary tract infections in the course of urolithiasis, reflux and/or glomerulonephritis accompanied by difficulty to treat arterial hypertension ( 1 , 12 - 17 ).

The specific group consisted of patients with bilateral nephrolithiasis. In our study, there were 2 patients with staghorn calculi, a significant inflammatory reaction that made the entire procedure difficult. Another group of patients consisted of those who had experienced previous rejection and removal of the graft. These patients had extensive scars on the abdomen, which determined the choice of retroperitoneal approach.

The use of the retroperitoneal approach that does not affect the peritoneal cavity in patients prior to the planned transplant appears to be a reasonable choice. However, the operation in dialysis patients requires a specific approach. First, we do not use the balloon to create retroperitoneal space, similarly to Doublet et al. ( 4 ). As a result, no patient suffered from damage to the peritoneum or other organs. In all 7 patients, the retroperitoneal space was created only with trocar and optics, without using balloon dilatation of the retroperitoneal space. We were prompted by reduced fat tissue in dialysis patients to perform such a manoeuvre. The optics allowed the manoeuvre to create the retroperitoneal space to be carried out safely under sight control. Time was also saved. We used three trocars on each side for the procedure. After the introduction of the trocars, the preparation was started from the visualization of the kidney cavity. First, the renal pelvis, artery and vein were localized, and then, the ureter was visualized. This is not a routine procedure. Usually, the procedure of removal of the kidney begins with the visualization of the ureter and, following it, until reaching the renal pedicle. The ureter of the inactive kidney can be very narrow and might not be easily localized, which occurred in this case. The use of modifications in the form of pelvis dissection in the first stage facilitated the location of the ureter, which was then easily dissected and cut off after closing the kidney vessels. Further preparation of the kidney did not differ from the routine procedure. The operation of bilateral simultaneous removal of the kidney in patients undergoing dialysis with extra-spinal laparoscopy does not cause a higher risk than in patients without dialysis. Our operations lasted from 2.5 to 3.5 hours in total in both types of patients. During this time, we had to change the position of the patients, which was the biggest inconvenience of the procedure. The method of the retroperitoneal approach in the prone position has also been described and makes it possible to perform the procedure without changing the patient’s position ( 9 - 11 ). This is a very interesting and remarkable proposition. However, in the work of Tanaka et al. ( 10 ), despite the lack of necessity to change the position of the patient, the operation time was much longer than ours, the operative time averaged 325min for the extirpative procedures (range 250-460 min) ( 10 ). Gundeti et al. ( 11 ) performed treatments in a shorter time of 110 to 180 min, on average 160 min, but in one patient, he was forced to convert due to peritoneal damage ( 11 ). Our procedures lasted from 150 to 240 min, on average 195 min, despite the need to change the position of the patient. We did not report any complications and did not have to convert in any of the cases. In our series of 7 patients, all patients underwent successful complete nephrectomy laparoscopically. In our study, the blood loss ranged from 100 to 250mL, average 142mL. The decrease in haemoglobin ranged from 0.1 to 1.4mg%, and no patients required transfusions. The average blood loss in the study of Tanaka was 281mL (range 15-739mL), and a patient required a transfusion ( 10 ). The result obtained by us likely results from a modification of the procedure, which deviates from the standard nephrectomy. This modification consisted of the following: resignation from the use of the balloon to create the retroperitoneal space and starting the preparation of the kidney from the cavity. We removed all the kidneys through the hole after removal of the 10mm trocar, after splitting it in the laparoscope sack. Morcellation did not significantly prolong the operation time, taking a maximum of 5 min. In most cases, we removed small kidneys from 5.8 to 8.2cm long. Two kidneys with a length of 10cm and the presence of staghorn calculi constituted a certain difficulty. The soft stones were crushed with Kocher’s forceps, to the extent that they could be removed through the hole after the 10mm trocar. Morcellation allowed us to avoid widening the hole to remove the tissue. In the case of bilateral nephrectomy, the only disadvantage of the access we used was the need to change the patient’s position. The inconvenience was compensated by an excellent view of the operating field, providing the opportunity to safely carry out the procedure. Similarly to other authors, we included oral intake on the first or second postoperative day ( 10 ). The technique of retroperitoneal access, in contrast to transperitoneal access, does not require preparation of the intestines, which allows quick return of peristalsis and immediate inclusion of oral intake. The omission of the peritoneal cavity is associated with a lower probability of damage to intraperitoneal organs, and previous abdominal operations do not affect the course of the procedure. In our case, graftectomy in 2 patients did not impair the procedure. One patient with transperitoneal dialysis, due to the use of retroperitoneal technique could continue this dialysis during the entire convalescence cycle. This is undoubtedly an advantage of this technique especially in peritoneal dialysis patients ( 11 , 18 , 19 ). In the transperitoneal approach, this cycle would have to be postponed up to 5 days ( 15 , 16 ).

Patients were hospitalized 2 days after surgery (except for 3 days in one patient with transperitoneal dialysis). Shorter stays have been described in the literature, even as short as 1 day ( 10 ) but in transperitoneal access 5.9 days ( 20 ). In our study, the hospitalization time of 2 days should be assessed as relatively short and acceptable in the context of patient safety and organizational conditions of the health care system.

A limitation of our work is the small number of patients, however, the qualification of nephrologists for simultaneous bilateral nephrectomy is also rare. Our goal was to demonstrate the safety and efficacy of the method of kidney removal through the retroperitoneal approach. Retroperitoneoscopic simultaneous bilateral nephrectomy is a well-tolerated and safe procedure for the patient. We did not report any intra or postoperative complications. The retroperitoneal approach allowed one patient to maintain peritoneal dialysis throughout the postoperative period. Furthermore, use of retroperitoneal approach in patients with indication for simultaneous removal of native kidneys gives the possibility of oral intake and, if necessary, transperitoneal dialysis on the first day. Laparoscopic bilateral nephrectomy followed by kidney transplantation is a safe and feasible alternative.

## CONCLUSIONS

The retroperitoneoscopic technique appears to be particularly attractive among numerous methods used in bilateral nephrectomy for patients undergoing dialysis. Retroperitoneal laparoscopic bilateral native nephrectomy is a safe and effective technique and allows for a short hospitalization and quick convalescence. The use of retroperitoneal access allows for preserving the peritoneal integrity and vessels for future vascular access.

The technique proposed by us, that is simultaneous bilateral nephrectomy with retroperitoneal approach, with the patient’s transposition and creation of retroperitoneal space, without the use of a balloon and beginning of the kidney preparation from the cavity side was safe and well tolerated in our patients. However, it should be emphasized that it requires considerable experience with laparoscopic surgery and strict adherence to several, described in our publication, technical points to ensure success. Compared to the literature data on laparoscopy in this setting, the retroperitoneoscopic nephrectomy can be considered the ideal approach for minimally invasive nephrectomy.
